# Stakeholder perspectives to inform adaptation of a hypertension treatment program in primary healthcare centers in the Federal Capital Territory, Nigeria: a qualitative study

**DOI:** 10.1186/s43058-021-00197-8

**Published:** 2021-08-30

**Authors:** Rosemary C. B. Okoli, Gabriel Shedul, Lisa R. Hirschhorn, Ikechukwu A. Orji, Tunde M. Ojo, Nonye Egenti, Kasarachi Omitiran, Blessing Akor, Abigail S. Baldridge, Mark D. Huffman, Dike Ojji, Namratha R. Kandula

**Affiliations:** 1 University of Nigeria, Nsukka, Nigeria; 2 University of Abuja Teaching Hospital Gwagwalada, Abuja, Nigeria; 3 Feinberg School of Medicine, Northwestern University, 420 E Superior, 6th Floor, Chicago, IL 60611 USA

**Keywords:** Hypertension, Implementation, Qualitative, Evidence-based, Primary care, Noncommunicable diseases

## Abstract

**Background:**

Implementing an evidence-based hypertension program in primary healthcare centers (PHCs) in the Federal Capital Territory, Nigeria is an opportunity to improve hypertension diagnosis, treatment, and control and reduce deaths from cardiovascular diseases. This qualitative research study was conducted in Nigerian PHCs with patients, non-physician health workers, administrators and primary care physicians to inform contextual adaptations of Kaiser Permanente Northern California's hypertension model and the World Health Organization’s HEARTS technical package for the system-level, Hypertension Treatment in Nigeria (HTN) Program.

**Methods:**

Purposive sampling in 8 PHCs identified patients (*n* = 8), non-physician health workers (*n* = 12), administrators (*n* = 3), and primary care physicians (*n* = 6) for focus group discussions and interviews. The Primary Health Care Performance Initiative (PHCPI) conceptual framework and Consolidated Framework for Implementation Research (CFIR) domains were used to develop semi-structured interviews (Appendix 1, Supplemental Materials) and coding guides. Content analysis identified multilevel factors that would influence program implementation.

**Results:**

Participants perceived the need to strengthen four major health system inputs across CFIR domains for successful adaptation of the HTN Program components: (1) reliable drug supply and blood pressure measurement equipment, (2) enable and empower community healthcare workers to participate in team-based care through training and education, (3) information systems to track patients and medication supply chain, and (4) a primary healthcare system that could offer a broader package of health services to meet patient needs. Specific features of the PHCPI framework considered important included: accessible and person-centered care, provider availability and competence, coordination of care, and proactive community outreach. Participants also identified patient-level factors, such as knowledge and beliefs about hypertension, and financial and transportation barriers that could be addressed with better communication, home visits, and drug financing. Participants recommended using existing community structures, such as village health committees and popular opinion leaders, to improve knowledge and demand for the HTN Program.

**Conclusions:**

These results provide information on specific primary care and community contextual factors that can support or hinder implementation and sustainability of an evidence-based, system-level hypertension program in the Federal Capital Territory, Nigeria, with the ultimate aim of scaling it to other parts of the country.

**Supplementary Information:**

The online version contains supplementary material available at 10.1186/s43058-021-00197-8.

Contributions to the literature

- Hypertension is a major threat to the well-being of people in Nigeria, and hypertension control is a priority of Nigeria’s National Multisectoral Action Plan on noncommunicable diseases.
- Interviews with patients and health professionals provided new insights into health system and community-level strengths and challenges to implementation of a multilevel hypertension program in primary health centers in Federal Capital Territory, Nigeria.
- Integration of community health workers into hypertension care and patient engagement, medication financing and availability, and primary care systems that support communication and coordination between health professionals, patients, and communities are keys to unlocking effective implementation, sustainability, and scale of the HTN program in Nigeria and global settings.

## Introduction

Hypertension, or high blood pressure, is a global public health problem which largely affects adults. It is a major risk factor for cardiovascular disease (CVD) [1, 2] and the third leading cause of global deaths [3], despite high-quality evidence demonstrating the efficacy and safety of blood pressure lowering medicines [2, 4]. An estimated 1.4 billion people worldwide have high blood pressure, but fewer than 15% of adults with hypertension worldwide have their blood pressure controlled to 140/90 or lower [5]. The burden of high blood pressure is higher in low- and middle-income countries (LMICs) where healthcare systems are often weaker than in high-income countries, leading to delays in diagnosis and low treatment and control rates [4, 6, 7]. Studies in African countries have shown the rising prevalence and burden of hypertension [1, 8]. In Nigeria, hypertension prevalence among adults ranges from 29 (95% CI, 25–33%) to 45% (95% CI, 44–46% )[1], yet awareness (14–30%), treatment (< 20%), and control (12%) rates among people with hypertension are estimated to be very low [1, 9, 10].

Multilevel, system-based interventions have been shown to significantly improve the prevention, detection, and control of hypertension [11, 12]. Such interventions include (1) behavioral changes, such as promoting healthy diets, active lifestyles, less alcohol consumption, and regular medication adherence at the individual level; (2) people-centered, accessible, and affordable primary care; and (3) a multidisciplinary, multisectoral collaboration between governments and civil society organizations [11, 13, 14]. Effective health system strategies for improving hypertension management, such as those developed by Kaiser Permanente Northern California and the World Health Organization through its HEARTS technical package include (1) patient registration with site-level audit and feedback, (2) automated blood pressure measurement, (3) simplified treatment protocols, including fixed-dose combination therapy, (4) team-based care including non-physician health workers to titrate medication and support self-management, and (5) a reliable supply of quality, affordable medicines [12, 15]. These models and intervention components could be adapted to Nigerian primary healthcare centers (PHCs) to improve hypertension diagnosis, treatment, and control rates as recommended as a priority area within the National Multisectoral Action Plan for the Prevention and Control of Noncommunicable Diseases [16]. However, successful adaptation of evidence-based programs for a new context requires understanding and incorporating the perspectives of patients, healthcare workers, and administrators about the facilitators and barriers to implementation pathways and program components.

We conducted this qualitative study as part of the Hypertension Treatment in Nigeria (HTN) Program, which aims to improve early detection, treatment, and control of hypertension in PHCs in the Federal Capital Territory. The HTN program includes multilevel, evidence-based strategies from the Kaiser Permanente Northern California (KPNC) model and World Health Organization (WHO) HEARTS package. These models were chosen because they have been demonstrated to be effective for lowering blood pressure [17] and improving hypertension control rates from 44 to 90% in unselected patients in the Kaiser Permanente Northern California health system [18]. The goal of this qualitative study was to understand (1) stakeholder perspectives on multilevel factors influencing blood pressure control and (2) how to adapt aspects of the Kaiser Permanente Northern California model and WHO HEARTS package for the primary healthcare system in the Federal Capital Territory, Nigeria.

## Methods

Briefly, the HTN program is a prospective, longitudinal type 2 hybrid implementation research study that is evaluating implementation and effectiveness of a multilevel, evidence-based implementation package using an interrupted time series design (NCT04158154). The HTN package includes (1) patient registration and empanelment, (2) standard treatment protocol, (3) encouragement of fixed-dose combination therapy, (4) team-based care, and (5) home blood pressure monitoring and health coaching. Prior to program implementation, the study team completed a mix of focus group discussions (FGDs) with patients and non-physician health workers and in-depth, individual interviews (IDIs) with PHC administrators and primary care physicians to inform cultural and contextual adaptation of the implementation package.

### Study setting and design

This formative study was conducted in 8 PHCs located in the Federal Capital Territory, Nigeria. The study design was cross-sectional qualitative that used a combination of FGDs (*n* = 10) and IDIs (*n* = 9) conducted between April 2019 and August 2019.

### Sampling

A purposive sample of participants was chosen based on their availability, experience, and knowledge of hypertension and their potential to provide perspectives needed to adapt the KPNC model and WHO HEARTS package to the target setting. Patients with high blood pressure (*n* = 8) and community health extension workers and nurses (*n* = 12) were selected for inclusion in the focus groups (3–4 participants from each of the 8 selected PHCs). PHC staff invited potential patients to participate in FGDs at their respective healthcare facilities. Each FGD lasted approximately 2 h, including the consent processes. IDIs were conducted with primary care physicians (*n* = 6) from 6 facilities and PHC administrators (*n* = 3) who were chosen from 3 of the 8 facilities and lasted 30 to 60 min. Demographic characteristics were obtained from all participants.

### Interview procedures and conceptual framework

**Fig. 1:**
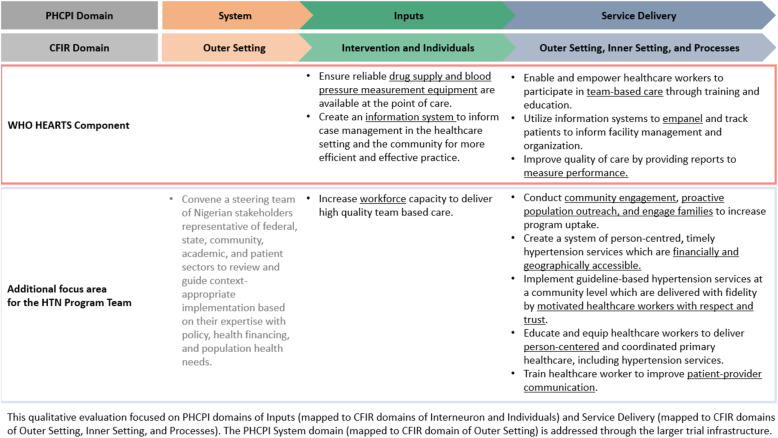
The PHCPI conceptual framework

We used a semi-structured interview guide for both FGDs and IDIs, which were developed using the Primary Health Care Performance Initiative (PHCPI) conceptual framework and mapped onto Consolidated Framework for Implementation Research (CFIR) main domains (Fig. 1) [19].

The IDIs and FGDs were performed on different days in the PHCs. We had two teams that engaged different groups in the respective PHCs. Written informed consent was obtained from each participant. Interviews were conducted by study investigators (NRK, RO, BA, GS, LRH, IO, NE) mainly in English language and colloquial English language (Pidgin), according to participants’ preferences. One patient FGD was conducted in Hausa (one of Nigeria’s three major languages). All FGDs and IDIs were audio recorded and professionally transcribed verbatim. The FGD in Hausa was translated and transcribed into English by a professional translator. All data and transcripts were anonymized and stored in a secured database at Northwestern University. The study was reviewed and approved by the Ethics Committee at the University of Abuja and the Institutional Review Board at Northwestern University.

### Data management and analysis

Dedoose qualitative software program (version 8.0. 35, SocioCultural Research Consultants, LLC, Los Angeles, USA) was utilized to analyze interview transcript. Two team members with qualitative research training and experience (RO, NRK) used an iterative process to code and analyze the transcripts with a directed content analytic approach [20] that was guided by the PHCPI mapped onto the CFIR matrix [19, 21]. The analysts used the multilevel factors from the PHCPI related to the implementation of hypertension care (Fig. 1) as codes during data analysis [22]. The researchers reached 100% consensus on all qualitative findings that are presented. The reporting of this study adheres to the Consolidated Criteria for Reporting Qualitative Research (COREQ) guidelines [23].

## Results

**Fig. 2:**
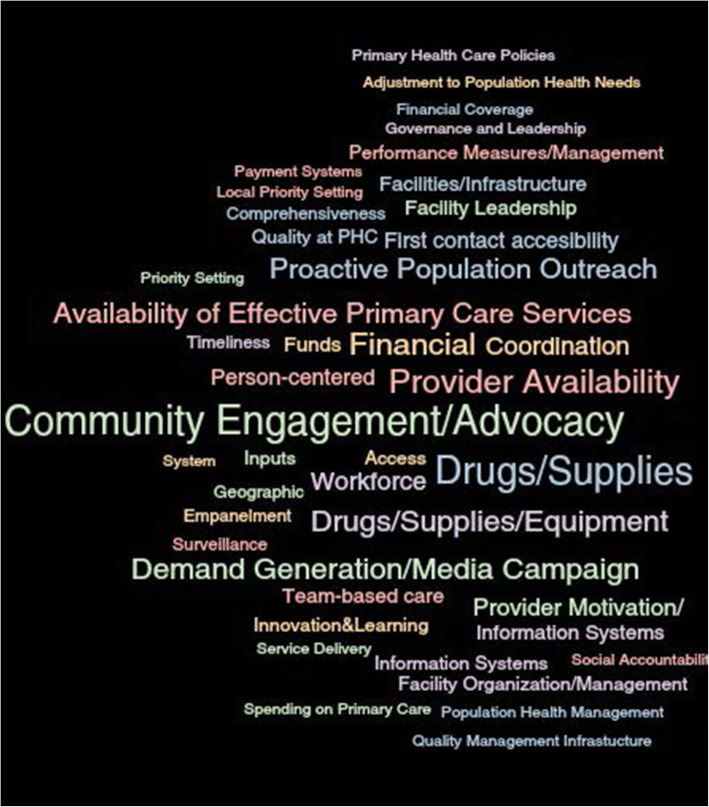
Word cloud of dominant themes in interviews

Characteristics of patients and non-physician health professionals are reported in Table 1. All patients had a diagnosis of hypertension, and the majority had a primary school education or less. Non-physician health workers and administrators had a college education or more and most were middle-aged. Data were organized into three major domains of the PHCPI and CFIR frameworks, and each domain contained several themes and subthemes (Fig. 1, Table 2). Several themes also overlapped with components of the World Health Organization HEARTS Technical Package, which are marked as red in Fig. 1. Two other dominant themes that emerged during interviews were about actors and process, i.e., patients, families, and healthcare providers, and the communication between them (Fig. 2).

**Table 1:** Focus group discussion and interview participants’ characteristics

	Patients (*n* = 8)	Non-physician health care workers (*n* = 12)	Administrator (*n* = 3)	Physician (6)
Age, mean (range)	61 (50–75)	44 (33–58)	58 (55–59)	43 (33–51)
Highest education level (*n*)	None (4), primary (2), secondary (1), college (1)	College (12)	College (2), postgraduate (1)	Postgraduate (6)
Occupation (*n*)	Self-employed (2), farmer (3), unemployed (1), other (2)	CHEW (9), CHO (1), nurse (2)	CHO, nurse physician (1 each)	Primary care physician (6)
HTN diagnosis	8	NA	NA	NA
On medication if diagnosed with HTN	8	NA	NA	NA

*CHEW* community health extension work, *CHO* Community Health Officer, *NA* not applicable

**Table 2:** Themes and quotes that emerged from formative interviews about the hypertension program in primary care clinics

Domains and themes	Quotes
**I. System (outer setting)**	
Ia. Improve quality of care by providing reports to measure performance	“Yes, ah, like for immunization, every month, after giving the report, we have a general meeting for only those who report those things so that you’ll know, so that you’ll know how far you are doing. We are even given categories. And I will like to take opportunity to tell you that this clinic is under grade one for the past ten years, in terms of our report.”
**II. Inputs (intervention and individuals)**	
IIa. Ensure reliable drug supply and blood pressure measurement equipment are available at the point of care	“Also drugs are free. Test is even free. So they will always come back. They will always; even they by themselves ma, will even go and _ cos I remember the first case we had of TB here, that was first treated here, he went and brought about five people.” “Drug Revolving Fund. What happen is that, government gives fund for [medicines].”
IIb. Create an information system to inform case management in the healthcare setting and the community for more efficient and effective practice	“Everyday, there is data that we do send. The patient that we have, we have register that we write, this is the patient we see. In fact, every month, every department gives their records, data.”
IIc. Increase workforce capacity to deliver high-quality team-based care Enable and empower healthcare workers to participate in *team-based* care through training and education	“If it is still going higher as we said, then we will do the treatment though depend on whom is on the seat. [inaudible] their doctors. We don’t treat, the doctors does [sic] the work. But if doctor is not on seat, then anyone of us, if it is the nurse or the CHEW that is available, then we go ahead and do the treatment.” “We are very confident if there is good training. They must be training so that everybody knows what is expected of him.”
**III. Service delivery (outer setting, inner setting, and processes)**	
IIIa. Create a system of person-centered, timely hypertension services which are financially and geographically accessible	“Of course, because somebody having malaria will prefer to go, you collect two paracetamols, collect ehh…you know, just few vitamins, they will mix it with antibiotics, and then they will just take and then they find out that they are okay. So they will prefer to go to such places, hundred naira or two hundred naira you go there. But by the time they are now coming here, you want to check BP, you want to check weight, you want to open card, and all those protocols, they see it as this is a delay. So, that thing can stop them from coming.”
IIIb. Conduct community engagement, proactive population outreach, and engage families to increase program uptake	“In community work, we have what we call mobilizers, we have town announcers, they go to inform them on some important issues if we need them to be here. And mostly, they encourage them to have the, what is it called, the digital BP apparatus, which they can use on their own without even struggling to come here. And we have to get some of their relatives, that can read and can understand better to help us through them to bring them to us or to even call us if need be. So those are the things we do.”
IIIc. Implement guideline-based hypertension services at a community level which are delivered with fidelity by motivated healthcare workers with respect and trust Train healthcare worker to improve patient-provider communication	“It’s useful because once the drugs, you put the drugs there, which means, any other person that comes, should follow the same pattern. So that there will be uniformity in the treatment of hypertension.” “Just like in HIV, we have standing order.” “When I was told that I have high blood pressure, I was told to take care of myself, I should always be happy, I should not be sad in my life, because this illness even if you are taking your medicines but you don’t have peace in your home, you won’t get better. They said it’s treatment is living in peace, and also I should take care of myself and also take my medicines.” “Well, they didn’t. Right there and then, I was told that I have hypertension, but they didn’t explain anything to me.”
IIId. Educate and equip healthcare workers to deliver person-centered and coordinated primary healthcare, including hypertension services	“Hundred percent because we can see they are coming up very well. Like the patients of TB, they can confidently come here and we’ll give them all the confidentiality they need; like the HIV, we make sure we take care of the confidential aspect of it. We make sure we give them the drugs that they need; we give them the counseling, all the advice that they need to be able to make a positive impact on them, and even to pro, to progress even in the program itself.”

### System (outer setting)

Few participants spoke about system-level factors that might influence implementation of the HTN Program. Separate from this qualitative study, the HTN team convened a steering committee of Nigerian stakeholders representative of federal, state, community, academic, and patient sectors to review and guide system-level adaptations based on their expertise with policy, health financing, and population health needs (Fig. 1).

### Inputs (intervention characteristics and individuals)

The most common themes related to intervention characteristics among patients, families, and providers were access to a reliable supply of quality and affordable blood pressure lowering drugs, workforce capacity and training, appropriate space and equipment to measure blood pressure, and funds to implement the HTN Program to address patient barriers.

#### Need for reliable drug supply and blood pressure measurement equipment

Both patients and healthcare workers agreed that affordability and accessibility to blood pressure lowering medicines would be essential to implementing and sustaining the HTN Program in the Federal Capital Territory public PHCs. For patients, issues focused largely on affordability and being able to obtain medication at the PHC, rather than a community pharmacy (Table 2). Participants also suggested using the drug revolving fund (DRF) model to address affordability wherein initial governmental funding for drug costs are used to subsidize reduced cost payments by patients, which in turn, helps to sustain the fund for subsequent drug purchasing. For example, one healthcare worker said,

*"The number one thing is that err, let the program be progressive, not saying that when at the end, when we have started the program, then maybe the availability, the supply of the drugs will be there frequently, but later on, it will not be there, you know it will discourage us. When patient comes, and discover they have high blood pressure and the drug is not there, so, like we the provider, in fact, I will not be more serious about it."*

#### Create information systems to inform case management

Most healthcare workers described some type of tracking system, which they viewed as helpful to empanel and track patients with a specific condition. One healthcare worker said:

*"It’s also good to have a register. In terms of, any patient we see, we record it, it’s also good for us to have. That’s for only hypertensive patients."*

Another healthcare workers stated:

*"Just like it is with TB (tuberculosis), we have a register for TB, that one is different. So if something like that could be brought into this issue, I think it will also assist."*

Registries for hypertension generally have not existed in Nigeria and need to be created for the HTN Program.

#### Increase workforce capacity to deliver high-quality team-based care

Some participants felt that staff numbers in certain clinics were adequate, but patients and some healthcare workers described low staffing as a major barrier to a successful implementation of the HTN Program. For example, one patient described several situations of low workforce availability:

*"Well, as for me, first of all, if it is possible, they should increase the number of personnel here, who would continue to manage this hospital. Because sometimes if you come you will see no one else but these [security] men here, and someone will say, ‘They have all gone home, until tomorrow’. Now the [blood] pressure has risen and you are told to go and come back tomorrow."*

From non-physician health care worker perspectives, they were willing to take on additional responsibilities to diagnose and manage hypertension, if they could receive additional trainings focused on diagnosing and managing hypertension. These trainings would also increase their motivation and confidence:

*"We are very confident if there is good training.*

*"There must be training so that everybody knows what is expected of him.*

Physicians thought that task-shifting was an appropriate solution to increase workforce capacity, and they stressed that non-physician healthcare workers needed ongoing training and supervision in hypertension management.

### Service delivery (outer setting, inner setting, and processes)

The strongest themes in this domain were about the importance of access to care, in terms of affordability, location, and timeliness (Table 2). Patients described long clinic waits, not being able to afford their medicines, and traveling long distances to reach the clinic. Participants agreed that if the clinics could supply drugs for free or at reduced cost, then this would increase the reach and effectiveness of the HTN Program.

The research team was also interested in understanding how quality improvement projects were integrated into current systems, and most participants used examples of how their clinic implemented quality improvement for communicable diseases or preventive services, and that this could be a model for noncommunicable diseases, including hypertension (Table 2).

#### Equip healthcare workers to deliver person-centered and coordinated primary healthcare

Both providers and patients said that high-quality primary care was demonstrated by being person-centered and that providers needed more training and resources to address patient needs beyond HTN. According to one healthcare worker:

*"I had two patients today for BP. One was, she doesn’t have anything to eat. So what I did, you know me ma, I don’t have much money. [Laughs]. You know, sometimes, it’s not sickness, but you need to give a helping hand……It’s not every day, but at least, you know someone that doesn’t have, even by mere looking at them, they don’t have, you support them."*

Coordination of care for more complex hypertension cases was also a concern for both patients and providers. Participants said it would be important to strengthen referral networks and communication between patients and providers when referrals happen. For example, a nurse manager said:

*"Because it is not easy for the person to come here and you are now telling him you are referring him to another place, so you really need to do a lot of talking, meaning that the person needs to be educated on why he needs to go that place, because it is expected that when they come here, everything here, should be done to them here."*

Physicians were particularly worried about blood pressure lowering medication side effects and complex cases. Some reported that communication between different team members and a referral process for complex cases would need to be created if the HTN Program were implemented in PHCs with non-physician healthcare workers.

#### Train healthcare worker to improve patient-provider communication

In addition to availability of providers and competence in diagnosing and treating hypertension, both patients and healthcare workers agreed that communication and trust were foundations of effective PHC services. In particular, participants noted that a key barrier was differences between patients and providers in explanatory models about hypertension and the treatment but that these differences could be bridged through effective communication. According to one health care worker:

*"The belief system of the people, most time when they come here, you know, sometimes we just don’t shut them up and say no it is not. So what we normally do, is okay, even if you believe that there’s … it is good to just try the medication and see how it goes. Let’s do it for two, three days, and then you’ll come back and let’s check. And some of them, you’ll find out that when they come back within that period, they’ll say ‘Ah nurse that your medicine really work, and then you can now continue with that. But most times, if you just shut them up and say ‘no there is nothing like that’, once they leave they don’t come back."*

Both non-physician healthcare workers and patients said that patient-healthcare worker communication should be improved as part of the HTN Program because it would help facilitate more effective hypertension management and control.

#### Conduct community engagement, proactive population outreach, and engage families

Health care workers felt that it was important for hypertension management and treatment to be community-centered by using active outreach and leveraging community structures to educate the public about hypertension, to create awareness of its risks and treatment options, and to increase demand for treatment. Proactive outreach was also viewed as important for blood pressure management and follow-up. For some clinics, this was already part of their strategy, as stated by one healthcare worker:

*"And we go out a lot. There is no case we don’t visit them at home, to find out how they are doing, how they are coping, what are their issues. So, with this I don’t think it is going to be an issue."*

Healthcare workers identified community leaders as important allies in implementing the HTN Program:

*"We have to go to the community leaders to inform them that this is what we are doing here. They will now give approval; they will help us to even talk to the people. We normally go, we have meetings with them."*

Led by health promoters, community area councils have existing structures where health education commonly takes place, and participants recommended that these could also be used as a place for people to measure their blood pressure (Table 2). This was perceived as an important, potential facilitator that could be implemented through the HTN program.

## Discussion

Interventions to detect and treat hypertension are urgently needed to curb the high incidence, prevalence, and burden of fatal and non-fatal cardiovascular disease and other hypertension-related conditions in Nigeria. Our study revealed that both patients and non-physician healthcare workers agreed that hypertension diagnosis and management should be a priority in PHCs in the Federal Capital Territory, and three important themes emerged to inform implementation and adaptation of the HTN program package at the PHC level. First, physicians and healthcare workers found it acceptable to have non-physician healthcare workers (specifically community health extension workers) diagnose and manage hypertension. Importantly, all stakeholders said that there must be a reliable and affordable supply of blood pressure lowering medications for successful program implementation. Third, stakeholders identified the need to strengthen many aspects of the primary healthcare system to make it more effective and person-centered. Our study is one of the first to use both the PHCPI and CFIR frameworks to conceptualize how a multilevel, system-based intervention needs to be adapted and implemented for hypertension control in a LMIC setting.

A key finding in the present study was that almost all participants felt comfortable with empowering non-physician healthcare workers (including community health extension workers) to diagnose, initiate, and conduct medication management with physician supervision for most patients with hypertension. Team-based care with non-physician health workers is part of both the Kaiser Permanente and WHO HEARTS models [15, 18] and is an intentional strategy to help the HTN program achieve success at scale. For the HTN Program to succeed, participants noted the need for adequate training and supervision of healthcare workers, which have not been previously provided. Based on this feedback, standardized training, continuing education, and supportive supervisions are integrated into the HTN Program, and these strategies have been identified by others as for programs to enhance the success and impact of programs with community health extension worker s[24].

Healthcare workers in the current study also said that having clinical practice guidelines and additional training on hypertension care would be beneficial, but that delivering high-quality hypertension care with fidelity would require additional support, such as standardized order sets, similar to what they used for treating HIV, as well as financial incentives to follow-up with patients and conduct home visits. Our findings provide more evidence on the specific health system setting, inputs, and processes, both financial and non-financial [25], that are needed to support the success of community health workers as an integral component of evidence-based hypertension care in PHC settings.

While patients and healthcare workers agreed that team-based care was acceptable and feasible, they said that care needed to be more person-centered and accessible and that communication between healthcare workers, referral networks, and patients could be improved. Participants said that that trust between healthcare workers and patients could be developed if they could address patients’ social needs as part of the HTN Program, and by working with the patient’s beliefs and attitudes about hypertension. Finally, healthcare workers recommended providing community-centered care, with home visits for monitoring and follow-up and utilizing community health committees already in existence in the area councils to provide hypertension education and screening. Community health extension workers often work in the communities where they live, and our study affirms that community linkage to PHC system through outreach, engagement, and referral networks may improve hypertension awareness, prevention, and treatment [24].

Because this intervention is being implemented in PHCs, there was an emphasis on how to strengthen the primary healthcare system for effective hypertension control. Healthcare workers and patients focused on strengthening the primary care system by improving first contact accessibility, provider availability and competence, coordination of care, and proactive community outreach. A narrative review of health system factors influencing hypertension care in several African countries also found that longer waiting times at health centers, limited capacity for adequate diagnosing and prescribing, and poor follow-up on non-adherent patients limited the capacity of some countries to manage and control hypertension [8]. Healthcare workers in our study suggested that there were already models in place for HIV and TB treatment that could be used to inform how PHCs manage hypertension. While healthcare workers were familiar with infectious disease-specific registries, they did not routinely have something similar for hypertension. However, registries for hypertension and other chronic diseases have not existed in Nigeria, and this study helped us identify the need for a HTN Program registry.

There was agreement among healthcare workers and patients in our study that outer setting and inner setting factors, such as financial barriers and stock outs of drugs, are major barriers to hypertension control. This is similar to what has been reported in other studies in Nigeria and many other settings [26–29]. Healthcare workers in our study suggested using a Drug Revolving Fund [14] to lower costs and to strengthen the blood pressure lowering medicine supply chain to avoid stock outs [30]. In another qualitative study with PHC workers and health insurance managers in western Nigeria, Kwara State, government-supported health insurance was perceived as an important facilitator for implementing high-quality hypertension care because it covered costs of care for patients and provided essential resources and incentives to implement high-quality hypertension care [31]. Our findings provide additional evidence that private-public partnerships and health system financing strategies are necessary to support HTN Program implementation and reduce the financial burden of hypertension management on individual clinics and patients.

Our study also found that the external environment or community-centered care, both in the form of home visits and provision of education and blood pressure monitoring in the community, were perceived to be important for improving hypertension control in the Federal Capital Territory. Several other studies suggest that leveraging the environment outside the clinic, through home visitations from non-physician healthcare workers and self-monitoring of blood pressure can lead to more effective hypertension management [32, 33]. In the Federal Capital Territory, Nigeria, implementing home and self-monitoring of blood pressure may require further context-specific adaptations. For example, participants in the current study noted that it may be more feasible to use community outreach locations as a centralized place to provide blood pressure monitoring and education, rather than people’s homes.

In a study of PHC healthcare workers and patients, Akinlua et al. found that there were differences in the knowledge and beliefs of health care workers and patients about the etiology and consequences of hypertension [34]. Similar to our study, patients held multiple beliefs about the causes of hypertension, many of them with cultural salience, and healthcare workers recognized the need to incorporate these cultural understandings into treatment plans. While our study was not designed to focus on healthcare worker and patient-level knowledge and beliefs, themes around patient-centered communication and respecting patient beliefs did emerge as important issues to address when implementing an evidence-based hypertension intervention in the PHC setting in the Federal Capital Territory, Nigeria.

Our findings have a number of limitations. First, the sampling was purposive and while we may not have captured the full diversity of patient and healthcare worker perspectives on hypertension, these data are broadly relevant to the sites of intervention within the HTN Program. Second, this is also a relatively small study conducted in the Federal Capital Territory, Nigeria, which does not necessarily reflect the PHC systems across the country, and it is unknown if the findings could be applied in other parts of Nigeria. We also did not interview administrators working at the state-level which may be one of the reasons our interviews did not have much information on system-level factors. However, we did conduct interviews and focus group discussions in 8 different PHCs to understand multilevel factors across the primary health system, and used the PHCPI and CFIR frameworks to provide a common set of themes and characteristics that could be considered when undertaking similar efforts in new settings.

## Conclusions

The Pan-African Society of Cardiology Hypertension Roadmap [35] recommends team-based care using simplified treatment guidelines, integration with existing services, and information systems to treat hypertension. These formative study results provide information on specific health system and community contextual factors that can support or hinder the implementation of effective, proven strategies within the primary care system of the Federal Capital Territory, Nigeria, and are directly aligned with the strategic priority areas within Nigeria’s National Multisectoral Action Plan for the prevention and control of noncommunicable diseases [16]. Integration of community health workers into hypertension care and patient engagement, medication financing and availability, and primary care systems that support communication and coordination between health professionals, patients, and communities are keys to unlocking effective implementation, sustainability, and scale of the HTN program in Nigeria and global settings.

## Supplementary Information

**Additional file 1.** Interview Guide for Administrators. Interview Guide for Patients. Interview Guide for Physicians.

## Data Availability

The datasets analyzed during the current study are not publicly available because the data collection as approved by the IRB did not include having them become publically available. The data can be made available to other researchers by contacting the corresponding author.
