# Supplementary material for: Stakeholder perspectives to inform adaptation of a hypertension treatment program in primary healthcare centers in the Federal Capital Territory, Nigeria: a qualitative study
Source: Implement Sci Commun. 2021 Aug 30;2:97. doi: 10.1186/s43058-021-00197-8 (PMC8404273; doi:10.1186/s43058-021-00197-8)
Supplement: Supplementary file 1 — Additional file 1. Interview Guide for Administrators. Interview Guide for Patients. Interview Guide for Physicians. [file 43058_2021_197_MOESM1_ESM.zip › Interview Guide for Administrators v1.1R1.docx]

**Formative Mixed Methods Implementation Package Development for the Transforming Hypertension Management in Nigeria Program**

Interview Guide

**Participants:** Administrators and Non-Physician Healthcare Workers

**Intervention, values, and perceived need**

1. Does your clinic diagnose, treat, or manage patients with hypertension?
   1. If no, what happens to patients with hypertension at your clinic? What does your clinic do with these patients?
   2. If yes, how big a problem is diagnosis, treatment, and management of hypertension in your clinic?
   3. *Probes: What have you seen that worked/not work? Why/why not?*
2. We are looking to try an intervention that includes patient registration, audit and feedback for quality and performance reporting, standard treatment orders and algorithms that prioritize fixed-dose combination and care provision led by community health extension workers. Tell me your thoughts about the feasibility of this intervention, including its individual components.
   1. *Probe: Tell me about the last time that you implemented a new intervention at this site. How hard would this be to implement?*
3. Tell me what this clinic needs to make this intervention accepted and implemented at your site.
   1. *Probes: What structural changes are needed for this to be successful? What resources are needed? Are there competing priorities right now? Would the patients accept this?*

**Relative advantage and self-efficacy**

1. Have there been any other programs for hypertension in your center before?
   1. If so, how does the intervention compare to other, similar existing programs in your setting like a TB or HIV program?
2. How confident would you be that this intervention would be effective at improving blood pressure control at this clinic?
3. How confident are you that this site and its team members, including you, can implement this intervention?
   1. *Probe: Why or why not?*

**Culture**

1. How hard has it been to introduce new interventions in your setting in the past?
   1. *Probes: What has helped? What has made it hard?*
2. How open will the leadership at your site be to this intervention?
   1. *Probe: If not positive, then what is needed to get their support?*
3. Who need to approve and support this intervention for it to work?
   1. *Probe: Other administrators, health care workers, patients, or other key stakeholders?*

**Implementation**

1. Describe how would you adopt the intervention to be implemented in your clinic.
2. What adaptations would be needed for your setting?
3. What kinds of information and materials about the intervention are needed for providers and patients?
   1. *Probe:.Do you have standing orders that you already use?*
   2. *Probe: Do you have educational aids for patients and providers? Are they effective?*
   3. *Probe: How can/should we raise awareness about this intervention and its benefits to the wider public?*
