# Supplementary material for: Stakeholder perspectives to inform adaptation of a hypertension treatment program in primary healthcare centers in the Federal Capital Territory, Nigeria: a qualitative study
Source: Implement Sci Commun. 2021 Aug 30;2:97. doi: 10.1186/s43058-021-00197-8 (PMC8404273; doi:10.1186/s43058-021-00197-8)
Supplement: Supplementary file 1 — Additional file 1. Interview Guide for Administrators. Interview Guide for Patients. Interview Guide for Physicians. [file 43058_2021_197_MOESM1_ESM.zip › Interview Guide for Patients v1.1R1.docx]

**Formative Mixed Methods Implementation Package Development for the Transforming Hypertension Management in Nigeria Program**

Interview Guide

**Participants:** Patients with hypertension

**Intervention, values, and perceived need**

1. How big do you think the problem of hypertension is to you?
   1. *Probes: What have you been doing to manage your hypertension?*
2. We are planning an interventional study that includes standard treatment for high blood pressure that emphasizes fixed-dose combination (combination of 2 drugs in 1 tablet), care provision led by community health extension workers, and home blood pressure monitoring. Kindly tell me how possible you think it is for this intervention method to be used in the treatment and management of your hypertension.
   1. *Probe: Have you ever taken a fixed-dose combination pill (one with two or three medication combined)?*
3. Tell me what this clinic needs to do so as to make this intervention acceptable to and implemented by you.
   1. *Probes: What should we do to make this possible?*

**Relative advantage and self-efficacy**

1. How does this intervention compare to how you currently manage your hypertension?
   1. *Probe: The intervention includes [show pictogram/visual] of the intervention.*
2. How confident would you be that this intervention would be effective at improving blood pressure control for you?
3. How confident are you that you could implement this intervention for your blood pressure management?
   1. *Probe: Why or why not?*

**Culture**

1. Has any new strategy or method being introduced to manage your hypertension?
2. How difficult was it to introduce this new strategy to manage your hypertension?
   1. *Probes: What has helped? What has made it hard?*
3. How open will you be to this intervention?
   1. *Probe: If not positive, then what is needed to get your support?*

**Implementation**

1. Are you willing to adopt this new intervention to manage your hypertension?
2. Describe how you will adopt the intervention for your hypertension management.
3. What adaptations would be needed for you?
4. What kinds of information and materials about the intervention do you need?
   1. *Probe: Share Resolve to Save Lives’s hypertension diagnostic and treatment algorithm. Share home blood pressure monitoring device and monitoring log.*
5. How can/should we raise awareness about this intervention and its benefits to the wider public?
