# Supplementary material for: Stakeholder perspectives to inform adaptation of a hypertension treatment program in primary healthcare centers in the Federal Capital Territory, Nigeria: a qualitative study
Source: Implement Sci Commun. 2021 Aug 30;2:97. doi: 10.1186/s43058-021-00197-8 (PMC8404273; doi:10.1186/s43058-021-00197-8)
Supplement: Supplementary file 1 — Additional file 1. Interview Guide for Administrators. Interview Guide for Patients. Interview Guide for Physicians. [file 43058_2021_197_MOESM1_ESM.zip › Interview Guide for PhysiciansR1.docx]

**Formative Mixed Methods Implementation Package Development for the Transforming Hypertension Management in Nigeria Program**

Interview Guide

**Participants:** Physicians

**Intervention, values, and perceived need**

1. Does your clinic diagnose, treat, or manage patients with hypertension?
   1. *Probe: What is your role in each of these tasks (direct provision, supervision etc)*
   2. *Probe: What have you seen that worked/not work? Why/why not?*
2. We are looking to try an intervention that includes patient registration, audit and feedback for quality and performance reporting, standard treatment orders and algorithms that prioritize fixed-dose combination and care provision led by community health extension workers. Tell me your thoughts about the feasibility of this intervention, including its individual components.
   1. *Probe: How are you currently treating patients with Hypertension? What drugs do you use or prescribe?*
   2. *Probe: What do you think about using Fixed Dose Combinations? About using a treatment algorithm?*
   3. *Probe: What do you think about CHEWs being involved in treatment and management of hypertension? How do you think CHEWs would need to be supported to ensure quality?*
3. Tell me what this clinic needs to make this intervention accepted and implemented at your site.
   1. *Probe: What structural changes are needed for this to be successful? What resources are needed?*
   2. *Probe: Are there competing priorities right now? Would the patients accept this?*
   3. *Probe: How do you think physicians would feel about supporting CHEWS to be leading this intervention? What role would you take/ What kind of supervisions is needed?*

**Relative advantage and self-efficacy**

1. Have there been any other programs for hypertension in your center before?
   1. If so, how does the intervention compare to other, similar existing programs in your setting like a TB or HIV program?
   2. *Probe: What were the roles of physicians, nurses and CHEWs?*
2. How confident would you be that this intervention would be effective at improving blood pressure control at this clinic?
   1. *Probe: What do you think of the proposed role of CHEWs in this intervention?*
3. How confident are you that this site and its team members, including you, can implement this intervention?
   1. *Probe: Why or why not?*

**Implementation**

1. What kinds of information and materials about the intervention are needed for providers and patients?
   1. *Probe:.Do you have standing orders that you already use?*
   2. *Probe: Do you have educational aids for patients and providers? Are they effective?*
   3. *Probe: How can/should we raise awareness about this intervention and its benefits to the wider public?*
